# Comparison of Pre-eclampsia Screening Algorithms in the First Trimester of Pregnancy Being Used in Thai Nguyen Province

**DOI:** 10.7759/cureus.59133

**Published:** 2024-04-27

**Authors:** Luong T Huong, Le Thi h Lan, Ha Thi V Hong, Nguyen v Cuong

**Affiliations:** 1 Obstetrics and Gynaecology, Thai Nguyen National Hospital, Thai Nguyen, VNM; 2 Biochemistry, Thai Nguyen National Hospital, Thai Nguyen, VNM

**Keywords:** biochemical biomarker, high-risk obstetrics, first trimester screening, preeclampsia-eclampsia, placenta growth factor (plgf)

## Abstract

Objective

To compare the rates of pregnancies in high-risk groups for preeclampsia recommended for aspirin prophylactic when screened using various common algorithms.

Methods

This cross-sectional study was conducted on 1726 pregnant women from 11 to 13 weeks six days of gestation receiving antenatal care at two hospitals: Thai Nguyen National Hospital and a hospital in Thai Nguyen Province from October 2022 to October 2023. All participants provided consent for the study. We collected maternal characteristics, obstetric history, mean arterial pressure (MAP), mean uterine artery-PI (UtA-PI), and placental growth factor serum (PLGF). Screening performance estimates were calculated using the Fetal Medicine Foundation (FMF) and National Institute for Health and Care Excellence (NICE) guidelines. All pregnant women in the study had their preeclampsia risk assessed using all three algorithms with two cut-off points. Our data was collected, entered and analyzed using SPSS software 20.0 (IBM Corp., Armonk, NY). Categorical data was reported as frequency and percentage. McNemar’s test was used for analyzing differences in the sizes of individual groups.

Results

In our study, the most common high-risk factor identified was the history of preeclampsia, 132 cases (7.6%). According to the NICE guideline, BMI ≥ 35 (kg/m²) is considered a moderate risk factor for preeclampsia. Several risk factors, such as BMI ≥ 35 kg/m² and history of diabetes mellitus type 1, were not present in any participants. Only one pregnant woman had chronic kidney disease (0.06%). Out of the 1726 pregnant women surveyed, the rates of high-risk preeclampsia were as follows: 9.9% (171 cases) based on algorithm 1; 10.8% (187 cases) based on algorithm 2 with a cut-off point > 1/100, 11.8% (203 cases) with a cut-off point > 1/150; 10.3 % (178 cases) based on algorithm 3 with a cut-off point > 1/100, and 11.6% (201 cases) with a cut-off point > 1/150. Among these algorithms, pregnant women in the high-risk preeclampsia group were advised to consider taking low-dose aspirin.

Conclusion

Screening for pre-eclampsia based on NICE recommendations resulted in a lower number of high-risk pregnant women requiring prophylactic aspirin use compared to other algorithms. This means that some pregnant women at risk of developing preeclampsia are not recommended to use aspirin as a preventive measure. Adding PLGF to the screening strategy will help us get closer to pregnant women who are truly at risk of progressing to preterm preeclampsia.

## Introduction

Preeclampsia is a pregnancy-specific disease, affecting 2.2-3% of all pregnancies [1,2]. The definition of preeclampsia has evolved over time. Its features are high blood pressure and endothelial dysfunction, leading to widespread end-organ injury. Preeclampsia is one of the main causes of maternal, fetal, and neonatal mortality, especially in low-income and middle-income countries [3]. In Vietnam, the prevalence of preeclampsia ranges from 2.84-3.8% [4,5], similar to rates in other parts of the world. For decades, scientists have been tirelessly searching for early screening and prevention methods to reduce the impact of this condition. In recent years, there has been a growing focus on predictive biomarkers for preeclampsia. Examples of these biomarkers include biochemical factors, placental growth factor serum (PLGF), and pregnancy-associated plasma protein A (PAPP-A). In cases of suspected preeclampsia, PLGF and PAPP-A levels are reduced in the first trimester [6,7]. PLGF is a vascular endothelial growth factor that is essential for the angiogenesis of the uteroplacental circulation, low levels of which precede the clinical manifestations of the disease. Numerous studies have demonstrated that screening for preeclampsia based on only one factor does not give results as good as combining many other factors. The algorithm proposed by the Fetal Medicine Foundation (FMF) has seen one of the most extensive validation worldwide. The FMF algorithm integrates maternal factors, obstetric history, physiology, and biochemistry. The Combined Multimarker Screening and Randomized Patient Treatment with Aspirin for Evidence-Based Preeclampsia Prevention (ASPRE) trial demonstrated that screening based on the FMF algorithms with the threshold of one in 100 predicted 90% of early preeclampsia, 75% of preterm preeclampsia and 41 of term preeclampsia, at a screen-positive rate of 10%. When utilized to determine which pregnant should be recommended aspirin for preeclampsia prevention, it notably reduces instances of preterm preeclampsia. Consequently, this reduction in preterm births leads to lower hospital expenses and decreased overall long-term financial and human costs associated with premature birth [8].

The FMF algorithm has been endorsed for preeclampsia screening by the FMF, the International Federation of Gynecology and Obstetrics (FIGO), and the Polish Society of Gynecologists and Obstetricians (PTGiP). However, there is a discrepancy in the recommended cut-off points. The FMF and PTGiP suggest utilizing a cut-off point of > 1/150 (FMF specifies this as suitable for the Caucasian population). On the other hand, The FIGO recommends a cut-off point of > 1/100 [9,10].

Thai Nguyen is a midland province in the northeastern region of Vietnam. In the past, preeclampsia screening has been conducted based on the National Institute for Health and Care Excellence (NICE) or FMF criteria, but the PLGF quantification technique has not been utilized. Recognizing the advantages of FMF’s preeclampsia screening approach, we have chosen to introduce PLGF quantification and incorporate it into FMF’s preeclampsia risk stratification screening algorithm.

## Materials and methods

Methodology

This is a cross-sectional study. Our research involved all pregnant women with a gestational age between 11 and 13 weeks and six days who attended prenatal check-ups at Thai Nguyen National Hospital and Thai Nguyen Hospital A and agreed to participate in the study. We excluded from our study the following cases: pregnant women with severe medical or mental conditions and those unable to provide accurate personal information. Or instances where the fetus has significant abnormalities, leading to a prediction that the pregnancy will not progress.

We gathered participant characteristics, obstetric history, and measurements such as mean arterial pressure, mean uterine artery pulsatility index (PI), and serum PLGF levels converted into multiples of the medians (MoM). These characteristics included age, BMI (body mass index), family history of preeclampsia, diabetes mellitus (DM) type 1 or 2, chorionic hypertension, systemic lupus erythematosus (SLE), antiphospholipid syndrome (APS), chronic kidney disease. Obstetric characteristics included the history of preeclampsia, method of conception, and single or multiple pregnancy. All pregnant women in the study had their preeclampsia risk assessed using all three algorithms with two cut-off points. We utilized model-based estimates to assess screening performance based on FMF and NICE guidelines. We called Algorithm 1: Screening with NICE guidelines(based on Maternal characteristics); Algorithm 2: Screening with FMF calculator (based on Maternal characteristics + UtA-PI + MAP) [10]; Algorithm 3: Screening with FMF calculator (based on Maternal characteristics + UtA-PI + MAP + PLGF) [10].

With algorithm 1, we had no cut-off point, because according to the NICE guidelines, they advised prophylactic aspirin, based on high-risk factors or more than one moderate-risk factor. With algorithms 2 and 3, we utilized the FMF calculator. After entering the information of the pregnant woman, the software calculated the risk. Depending on each cut-off threshold, we identified the group of pregnant women who should be prescribed aspirin to prevent preeclampsia. We have selected 2 cut-off points for the preeclampsia risk group, for whom aspirin should be administered: Cut-off point > 1/150 according to the FMF and the PTGiP recommendations and Cut-off point > 1/100 according to the FIGO recommendations. The FMF algorithm, which is on the third screen above, has been proven to have a superior screening effect by the ASPRE trial [8]. Therefore, we have chosen to conduct screenings locally [10].

Variables and data analysis

Our data was collected, entered and analyzed using SPSS software, version 20.0 (IBM Corp, Armonk, NY). Categorical data was reported as frequency and percentage. McNemar’s test was used for the analyzing differences in the sizes of individual groups, the result is said to be statistically significant if a p-value is less than 0.05.

Ethical approval

This study was approved by the Ethical Committee of Thai Nguyen National Hospital with approval number IRB: 534/CN-TWTN. Our study has also been approved by the Department of Science and Technology of Thai Nguyen province and Thai Nguyen hospital A. Pregnant women in our study were introduced to and informed about the study objectives and participated (signed consent form). Participants could withdraw at any time, and their information was kept confidential.

## Results

There were 1726 pregnant women from October 2022 to October 2023 found to be eligible to participate in our study. In Table 1, according to the NICE guidelines, BMI ≥ 35 (kg/m²) is considered a moderate risk factor for preeclampsia; however, none of the women in our study presented this risk factor. Regarding the participants’ BMI, the median was 19.6 (with a range of 16.8-31.2). Only one pregnant woman had chronic kidney disease (0.06%). The high-risk factor that was the most popular in our study was the history of preeclampsia in 132 pregnant women (7.6%).

**Table 1:** Risk factors of pregnant women in our study BMI: Body Mass Index, DM type 1: Diabetes Mellitus type 1, DM type 2: Diabetes Mellitus type 2, SLE: Systemic Lupus Erythematosus, APS: AntiPhospholipid Syndrome.

Characteristic	N(%)
	Median (IQR)
Age≥ 40 years	5 (0.3%)
	26 (17 - 44)
BMI ≥ 35 (kg/m²)	0 (0%)
	19.6 (16.8 – 31.2)
History of preeclampsia	16 (0.9%)
Medical history	
Chorionic hypertension	16 (0.9%)
DM Type 1	0 (0%)
DM Type 2	5 (0.3%)
SLE/APS	7 (0.4%)
Chronic kidney disease	1 (0.06)
History of preeclampsia	132 (7.6%)
Family history of preeclampsia	88 (5.1%)
Method of Conception	
Natural	1615 (93.6%)
Ovulation drugs	14 (0.8%)
In-vitro fertilization	97 (5.6%)
Obstetric history	
Nulliparous	741 (42.9%)
Parous	985 (57.1%)
Multiple pregnancy	19 (1.1%)
Total pregnant women	1726 (100%)

Table 2 displays the number of the high-risk groups for preeclampsia, based on different algorithms. Our analysis revealed no statistically significant differences in the numbers of pregnant women in the high-risk group when comparing algorithm 2 and algorithm 3 (with or without using PLGF) at the same cut-off point (p=0.894 và p= 0.2). However, a statistically significant difference was observed between algorithm 1 and algorithm 3 (cut-off point 1/150) based on the results of the McNemar test, with p= 0.018.

**Table 2:** Compare screening algorithms with two cut-off points MAP: Mean Arterial Pressure, UtA-PI: Uterine Artery-Pulsatility Index, PLGF: Placental Growth Factor, Algorithm 1: Screening based on Maternal characteristics (NICE guideline), Algorithm 2: Screening based on Maternal characteristics + UtA-PI + MAP (use FMF calculator), Algorithm 3: Screening based on Maternal characteristics + UtA-PI + MAP + PLGF (use FMF calculator), FMF: Fetal Medicine Foundation, MAP: Mean arterial pressure.

Method of screening	Comparison of detection by two methods	P(value)
Algorithm 1 vs Algorithm 3 (cut-off > 1/100)	171 vs 178	0.644
Algorithm 1 vs Algorithm 3 (cut-off > 1/150)	171 vs 201	0.018
Algorithm 2 vs Algorithm 3 (cut-off > 1/100)	187 vs 178	0.894
Algorithm 2 vs Algorithm 3 (cut-off > 1/150)	203 vs 201	0.2

Table 3 shows the number of cases in the preeclampsia high-risk group. They were advised to take a low dose of aspirin daily. It can be seen that the number of pregnant women needing prophylactic aspirin based on algorithm 1 (the algorithm is simply based on maternal characteristics) is the smallest. Meanwhile, based on algorithms 2 and 3 with a cut-off point of > 1/150, the number of pregnant women who needed to take aspirin daily is the largest.

**Table 3:** The number of pregnancies were recommended to take prophylactic aspirin based on the algorithms MAP: Mean Arterial Pressure, UtA-PI: Uterine Artery-Pulsatility Index, PLGF: Placental Growth Factor, Algorithm 1: Screening based on Maternal characteristics (NICE guideline), Algorithm 2: Screening based on Maternal characteristics + UtA-PI + MAP (use FMF calculator), Algorithm 3: Screening based on Maternal characteristics + UtA-PI + MAP + PLGF (use FMF calculator), FMF: Fetal Medicine Foundation, MAP: Mean arterial pressure.

	Recommended to take Aspirin (%)
Cut-off > 1/100	
Algorithm 2	187 (10.8%)
Algorithm 3	178 (10.3%)
Cut-off > 1/150	
Algorithm 2	203 (11.8%)
Algorithm 3	201 (11.6%)
Algorithm 1	171 (9.9%)
Total number of pregnant women	1726 (100%)

## Discussion

Our study was conducted at the time when PLGF testing was first implemented in Thai Nguyen. Previously, we screened for preeclampsia risks based on NICE guidelines. In some cases where pregnant women already had PAPP-A, we used the FMF calculator. However, the traditional approach to identifying the high-risk group for preeclampsia, benefiting from prophylactic aspirin use, relies on risk factors derived from maternal demographic characteristics and medical history. This method, however, only identifies around 40% of preterm preeclampsia cases, with a false-positive rate (FPR) of 10%. The screen-positive rate using the NICE method was 10.3%, and the detection rate for all preeclampsia cases was 30.4%, with a higher rate of 40.8% for preterm preeclampsia. Compliance with the NICE recommendation that women at high risk for preeclampsia should take low-dose aspirin from the first trimester until the end of pregnancy was only 23%. The detection rate (DR) of the combined test for all preeclampsia was 42.5%, which was superior to that of the NICE guideline by 12.1% (95% CI, 7.9-16.2%). In screening for preterm preeclampsia based on a combination of maternal factors, MAP, and PlGF, the DR was 69.0%, outdoing the NICE guideline by 28.2% (95% CI, 19.4-37.0%). Moreover, if UtA-PI was added, the DR improved to 82.4%, surpassing that of the NICE method by 41.6% (95% CI, 33.2-49.9%) [11]. After the trial results were published, the preeclampsia screening model in various countries has changed, including Vietnam. In our efforts to minimize the risks associated with preeclampsia and eclampsia, we have incorporated PLGF into our first-trimester screening program.

In Table 1, we provide statistics on the proportion of risk factors in the study population. These risk factors were developed according to NICE recommendations, which we have previously used to screen for preeclampsia. NICE advises pregnant women with any high-risk factors or more than one moderate risk factor for preeclampsia to take 75mg to 150mg of aspirin daily from 12 weeks until the birth of the baby. High-risk factors identified by NICE include a history of preeclampsia, chronic kidney disease, systemic lupus erythematosus or antiphospholipid syndrome, type 1 or type 2 diabetes, and chronic hypertension. Moderate risk factors include first pregnancy, age 40 years or older, a pregnancy interval of more than 10 years, BMI of 35 kg/m² or more at the first visit, a family history of preeclampsia, and a multi-fetal pregnancy [12]. Thai Nguyen is one of the provinces with large industrial parks that have thousands of workers. Therefore, the majority of pregnant women in our study belonged to the young age group, and the obesity rate of women in our study is low. The criteria for determining risk factors, as recommended by NICE, are for women in European countries. There is still a lack of research on whether the application in Vietnam is as effective as in European countries. In our study, the most common high-risk factor is a history of preeclampsia, with 132 (7.6%) pregnant women affected. In 2020, a study by author Tran Manh Linh showed that chronic hypertension increases the risk of preeclampsia (OR = 16.72, 95% CI = 6.96 - 40.18 ) [4].

Our study found that the difference in the number of pregnant women in high-risk groups when screened with two algorithms (algorithm 3, which involves the addition of PLGF to algorithm 2) was not statistically significant at both cut-off points of >1/100 and > 1/150. The main reason for the lack of differences is the fact that some women were classified as high risk for preeclampsia when we used different algorithms. Therefore, when comparing differences between the groups that utilized algorithm 3 and algorithm 2, most were found to contain the same numbers of women, resulting in a lack of statistical significance. It is worth noting in our study that when comparing the number of pregnant women in the high-risk groups screened by algorithm 1 and algorithm 3, the difference is statistically significant with the cut-off point of > 1/150 (p=0.018). However, with the cut-off point of >1/100, the difference is not statistically significant (p=0.644). As we know, the NICE method treats each maternal factor as a separate screening test. In the FMF method, the use of a multivariable logistic model to define the prior risk attributes the appropriate relative importance to each maternal factor and allows for the estimation of the patient-specific risk of preeclampsia requiring delivery before a specified gestation [10]. The prior risk can then be adjusted according to the results of biophysical and biochemical testing. In our study, the prevalence of pregnant women who were in the high-risk group of preeclampsia based on algorithm 3 with a cut-off point > 1/100 is 10.3% (178 women). This prevalence in the study of Tran Manh Linh in Hue province in 2020 is 21.9%. As we know, the prevalence of the disease and the characteristics of the population can influence the effectiveness of screening tests [8]. The study of Tran Manh Linh utilized a combination of maternal factors + MAP + Uta-PI + PAPP-A, which is different from ours [4].

At present, aspirin is the only therapy with robust evidence supporting its use to reduce the risk of preeclampsia in high-risk women. Current recommendations are to take low-dose aspirin (75-150mg) as prophylaxis from 12 weeks to 36 weeks of gestation. When taken before 16 weeks gestation, low-dose aspirin has a modest but consistent effect, estimated to reduce the risk of preeclampsia by approximately 10% [8]. In our study, when using algorithm 3 for screening, more pregnant women were recommended to take prophylactic aspirin compared to algorithm 1. However, we did not notice a significant difference in the number of pregnant women requiring aspirin between screening based on algorithm 2 and algorithm 3. Essentially, incorporating PLGF into algorithm 2 and adjusting the FMF calculator did not result in a change in the quantity of aspirin needed. A study conducted at University College London Hospital National Health Service (NHS) Foundation involving 5957 pregnancies showed that 12.8% and 15.9% of women tested positive for the development of preeclampsia using The NICE and FMF methods, respectively. Among women at high risk for preeclampsia screened according to The NICE guidelines, aspirin was not prescribed in 25% of cases. The utilization of the FMF algorithm resulted in seven fewer instances of preterm preeclampsia, cost savings of £9.06, and a QALY (Quality Adjusted Life Years) gain of 0.00006 per pregnancy screened [13].

In Vietnam, first-trimester screening for preeclampsia based on the FMF method with maternal characteristics + MAP + Uta-PI + PAPP-A is most common due to the availability of PAPP-A in chromosomal screening assays. However, the PLGF algorithm is characterized by higher efficiency [6,11]. Adding PAPP-A to the algorithm recommended by The FMF, The FIGO, and the PTGiP does not change the screening effectiveness in detecting women at risk of preeclampsia [9,14,15].

Wishing to help pregnant women as much as possible, we try to find the best screening algorithm for the Vietnamese population. Our study suggests including PLGF in the preeclampsia screening algorithm for the first trimester of pregnancy.

 Limitations

Due to limited resources, our study could not follow pregnant women until the end of pregnancy. In the future, we hope to have a larger study. We will start screening in the first trimester and continue until the end of pregnancy, which will help us evaluate the effectiveness of screening algorithms more accurately.

## Conclusions

Screening for pre-eclampsia based on NICE recommendations resulted in a lower number of high-risk pregnant women requiring prophylactic aspirin use compared to other algorithms. PLGF should be included in the preeclampsia screening algorithm in the first trimester of pregnancy to enhance the effectiveness of prophylactic treatment with aspirin.

## References

[1] Khan B, Allah Yar R, Khakwani AK, Karim S, Arslan Ali H (2022). Preeclampsia incidence and its maternal and neonatal outcomes with associated risk factors. Cureus.

[2] Yang Y, Le Ray I, Zhu J, Zhang J, Hua J, Reilly M (2021). Preeclampsia prevalence, risk factors, and pregnancy outcomes in Sweden and China. JAMA Netw Open.

[3] Souza JP, Gülmezoglu AM, Vogel J (2013). Moving beyond essential interventions for reduction of maternal mortality (the WHO Multicountry Survey on Maternal and Newborn Health): a cross-sectional study. Lancet.

[4] Linh TM Studying the results of screening for preeclampsia - eclampsia using PAPP-A test, uterine artery doppler and the effectiveness of preventive treatment. http://univ.edu.vn/newsmultidata/files/Luan%20van%20NCS/Tran%20Manh%20Linh/1-TMLinh-toan-van-luan-an.pdf.

[5] Vinh TQ, Yucheng G, Huy NVQ, Duc VV, Diem Thu NT, Linh TM Study on the effectiveness of preventive treatment of preeclampsia - eclampsia with calcium in pregnant women at high risk of preeclampsia - eclampsia (Article in Vietnamese). Vietnamese Journal of obstetrics and gynecology.

[6] O’Gorman N, Wright D, Syngelaki A. Competing risks model in screening for preeclampsia by maternal factors and biomarkers at 11-13 weeks gestation. Am J Obstet Gynecol.

[7] O'Gorman N, Wright D, Poon LC (2017). Multicenter screening for pre-eclampsia by maternal factors and biomarkers at 11-13 weeks' gestation: comparison with NICE guidelines and ACOG recommendations. Ultrasound Obstet Gynecol.

[8] Rolnik DL, Wright D, Poon LC (2017). ASPRE trial: performance of screening for preterm pre-eclampsia. Ultrasound Obstet Gynecol.

[9] Poon LC, Shennan A, Hyett JA (2019). The International Federation of Gynecology and Obstetrics (FIGO) initiative on preeclampsia (PE): a pragmatic guide for first trimester screening and prevention. Int J Gynaecol Obstet.

[10] (2024). Assessment of risk for preeclampsia. https://fetalmedicine.org/research/assess/preeclampsia/background%3E,%20accessed:%2003/22/2024.

[11] Tan MY, Wright D, Syngelaki A (2018). Comparison of diagnostic accuracy of early screening for pre-eclampsia by NICE guidelines and a method combining maternal factors and biomarkers: results of SPREE. Ultrasound Obstet Gynecol.

[12] NICE. NICE. (2024). Hypertension in pregnancy: diagnosis and management. https://www.nice.org.uk/guidance/ng133/chapter/Recommendations.

[13] Nzelu D, Palmer T, Stott D (2024). First trimester screening for pre-eclampsia and targeted aspirin prophylaxis: a cost-effectiveness cohort study. BJOG.

[14] Tousty P, Czuba B, Borowski D (2022). Effectiveness of different algorithms and cut-off value in preeclampsia first trimester screening. J Pregnancy.

[15] Mazer Zumaeta A, Wright A, Syngelaki A, Maritsa VA, Da Silva AB, Nicolaides KH (2020). Screening for pre-eclampsia at 11-13 weeks' gestation: use of pregnancy-associated plasma protein-A, placental growth factor or both. Ultrasound Obstet Gynecol.

